# Naturalistic outcome of treatment of psychosis by traditional healers in Jinja and Iganga districts, Eastern Uganda – a 3- and 6 months follow up

**DOI:** 10.1186/1752-4458-6-13

**Published:** 2012-09-08

**Authors:** Catherine Abbo, Elialilia S Okello, Seggane Musisi, Paul Waako, Solvig Ekblad

**Affiliations:** 1 Department of Psychiatry, College of Health Sciences, Makerere University, P.O Box 7072, Kampala, Uganda; 2 Department of Pharmacology and Therapeutics, College of Health Sciences, Makerere University, Box 7072, Kampala, Uganda; 3 Department of Learning, Informatics, Management and Ethics (LIME), Karolinska Institutet, SE-171 77, Stockholm, Sweden

**Keywords:** Psychosis, Treatment outcome, Traditional healers, Uganda

## Abstract

**Objective:**

To determine the naturalistic outcome of treatment of psychosis by traditional healers in Jinja and Iganga districts of Eastern Uganda.

**Method:**

A cohort of patients with psychosis receiving treatment from traditional healers’ shrines were recruited between January and March 2008 and followed up at three and six months. The Mini International Neuropsychiatry Interview (MINI Plus) was used for making specific diagnosis at the point of contact. For specific symptoms, Positive and Negative Symptom Scale (PANSS), Young Mania Rating Scale (YMRS) and Montgomery Asberg Depression Rating Scale (MADRS) were used to measure severity of schizophrenia, mania and psychotic depression, respectively. The Clinical Global Impression (CGI) and Global Assessment of Functioning (GAF) were used for objective assessments. The Compass Mental Health Index measured well being. Mean scores of the scales were computed using one way ANOVA for independent samples. Associations between outcome and categorical variables were examined at bivariate and multivariate levels.

**Results:**

All the symptom scales had a percentage reduction of more than 20% at three and six months follow up. The differences between the mean scores of the scales at baseline and 3 months, baseline and 6 months, and 3 and 6 months were all significant (P < 0.0001). The post test for pair wise comparisons, the Tukey HSD (Honestly Significant Difference) test was also all significant at P < 0.01 except for MADRS where there was no significant difference between 3 and 6 months for depression severity. Over 80% of the participants used biomedical services for the same symptoms in the study period. At 3 months follow up, patients who combined treatment were less likely to be cases (P = 0.002; OR 0.26 [0.15-0.58]), but more likely to be cases at 6 months follow up (P = 0.020; OR 2.05 [1.10-3.189]). Being in debt was associated with caseness both at 3 and 6 months.

**Conclusion:**

This study suggests that there may be some positive effects for patients with psychosis who combine both biomedical services and traditional healing**.** Further research in the area of naturalistic outcome of traditional healing is necessary**.**

## Background

The use of Traditional healing (TH) and Complementary and Alternative Medicine (CAM) is widely acknowledged and growing both in low and high income countries. In North America, Western Europe and other developed regions, an increasing number of patients are seeking out CAM practitioners for mental health care [1]. The use of CAM approaches is significantly greater among individuals who meet DSM-IV criteria for any psychiatric disorder than the general population [2]. In Africa, traditional healing is deeply embedded in the African traditional beliefs [3,4]. Because of what the community culturally perceives as the causation of severe mental illness, they have close relationship with traditional healers who often share the same community and culture.

Traditional medicine has been stated to be the pillar of health care in many developing countries [5]. According to the WHO, about 60-80% of the population in Sub Saharan Africa depend primarily on traditional systems of health care [6]. It had been suggested that to provide primary health care for all by the year 2000, in line with the Alma-Ata declaration, traditional medicine had to be accorded equal recognition as Conventional Western Medicine (CWM) [5]. It is clear that this goal has not been achieved and that health discrepancies have even widened since that time [7]. The reason for failure could be lack of the equal recognition and status of traditional healing to CWM. This in turn could probably be due to lack of outcome research in traditional healing. There is lack of evidence for effectiveness of traditional healing as there is paucity of randomized controlled trials of CAM [8]. Any available evidence relates mainly to depression and anxiety disorders with hardly any literature or evidence for psychosis [9-11]. This has created a public health challenge in terms of policy, safety, efficiency, efficacy, quality, access, training and rational use [12,13]. Faced with the challenge of evidence-based medicine, those who argue for the collaboration with traditional healers are often challenged with the question: “Does it work?” The lack of evidence is partly due to the difficulty of conducting evaluations of the complex social interventions typically deployed within traditional healing practices [14]. The important practical problem for researchers is attempting to reconcile methods and clinical evidence supporting non-conventional treatment with the experimental biological processes posited by the Conventional Western Medicine (CWM). Yet it is vital that practitioners of CWM become more knowledgeable about TH/CAM methods, possible benefits and limitations. By doing so, CWM practitioners will not only be able to serve as more useful sources of information to their patients and advise them appropriately but also give the much needed information to policy makers on the role of traditional healing practices in planning community mental health care [14].

In this paper, we present the results of a three and six month follow up of subjects who were identified as having a psychosis (schizophrenia, mania and psychotic depression) and treated by traditional healers. Our main objectives were to determine clinical outcome in terms of symptom control, subjective well being and social functioning of the patients seeking help from traditional healers and factors that may be associated with naturalistic outcome of treatment in two districts in Uganda. These results may give more insight in the role of traditional healers in mental health care in Uganda.

## Methods

This was a prospective cohort study carried out in two districts of Jinja and Iganga in Eastern Uganda. This cohort and its recruitment has been described in previous studies [15-17]. In summary, a total of 400 patients aged 18 years and above attending traditional healers’ shrines in Jinja and Iganga districts were recruited consecutively between the months of January and March 2008. The description of the selection of traditional healers’ shrine has been described in previous publications [15-17]. Those who scored six and above on the first 20 questions of SRQ 25 [15] and those who answered yes to any of the four questions that screened for psychosis were administered the MINI plus, a diagnostic instrument to make a psychiatric diagnosis [18]. Of the 387 respondents analyzed, 115(29.7%) had psychosis i.e. Schizophrenia n = 28, psychotic depression n = 46 and mania n = 41). They all agreed for a follow up at three and six months.

Four Psychiatric Clinical Officers working in Jinja and Iganga districts had training in making assessments using the rating scales and they rated the patients.

After complete description of the study to the subjects, written and verbal informed consent was obtained from the patients and their relatives. The patients were assessed at first point of contact and the ratings given to the principal investigator (first author) and this set of ratings were not available to the raters when they made the second and third assessment. This was to make the different assessments as independent from each other as possible.

Cases were interviewed by the research assistants with instruments that measure symptom severity as described in the section below:

1) schizophrenia

### The Positive and Negative Symptom Scale for Schizophrenia (PANSS)

The PANSS is a 30-item scale in which each item severity is rated on a Likert scale ranging from 1 (absent) to 7 (extreme). PANSS allows for separate analysis of positive symptoms, negative symptoms and general psychopathology. The PANSS provides a comprehensive rated assessment of symptoms of schizophrenia. Thus, the minimum total score for a patient who has no symptoms is 30 whatsoever and 210 for the maximum total score [19,20]. The PANSS has been demonstrated to be both reliable and valid and to have a high internal consistency [21]. It has been used world wide including in Uganda [22].

2) Mania

### Young Mania Rating Scale (YMRS)

The Young Mania Rating Scale has 11 items and is based upon the patient’s subjective report of symptom experience over the preoceding 48 hours. Additional information is based upon the clinical observations made during the course of the clinical interview. There are four items that are graded on 0 to 8 scale (irritability, speech, thought content, and disruptive/aggressive behaviour), while the remaining seven items are graded on a 0 to 4 scale. Score range is 0–60. A score of 20 is taken as indicative of illness [23].

3) Psychotic depression

### The Montgomery and Asberg Depression Rating Scale (MADRS)

The Montgomery and Asberg Depression Rating Scale (MADRS) rating is based on clinical interview ranging from broadly phrased questions about symptoms to more detailed ones. This allows for a precise rating of severity. Relevant clues as well as information from other sources can be used as a basis for rating in those patients who cannot communicate/speak. The scale has a total number of 10 items scored on a 0-6 Likert scale. Total score range is 0–60, the cut-off score is 10 with score of 10–17 indicating mild, 18-34 moderate and 35–60 severe depression [24]. A score of 9 and below indicates remission [25]. The MADRS has been used in Ugandan setting [26].

In addition, for all patients, the following instruments were administered to assess clinical outcome:

### The COMPASS mental health index

This is a brief self-reporting measure consisting of current life functioning, subjective well being and current symptoms that are scored and summed up to create an overall Index. This includes assessment of general mental health status, patient characteristics and outcome of care from individuals receiving treatment for any mental disorder [27]. The items of the scale are organized into subscales: subjective well being has 4 items, symptoms have 33 items, and overall functioning has 17 items. This study used only the subscale of subjective well being because this subscale has been validated in a Ugandan setting [26] and the symptoms and overall functioning was measured by other instruments. Subjective well being was scored over a 5 point Likert response scale as follows: 1 = not at all; 2 = a little bit; 3 = moderately; 4 = quite a bit; 5 = extremely. The scoring range is from 4 to 20. A response of 3, 4 or 5 was taken to mean better well being while a response of 1 and 2 as poorer state of life [28]. Thus, total scores of 4 = very poor; 8 = poor; 12 = fair; 16 = good and 20 = excellent indicated the degree of subjective well being and satisfaction with life.

### The Global Assessment of Functioning (GAF) scale

The GAF values ranges from 0 (most severe functional impairment) to 100 (least severe functional impairment) with 10 anchor points at equal intervals. Each interval of the GAF is accompanied by a behavioral descriptor ranging from “superior functioning in a wide range of activities with no symptoms to persistent danger of severely hurting self or other or persistent inability to maintain minimal personal hygiene [29].” Functioning according to GAF was rated by trained research assistants on the basis of information gathered from the patients at the traditional healer’s shrine. GAF assessments referred to the current symptoms. For the purposes of this study, GAF was classified in the following ways basing on previous studies [30,31]: GAF > 70 = Normal functioning; GAF 51–70 = Mild functional impairment ; GAF 31- 50 = moderate functional impairment; and GAF≤ 30 = severe functional impairment. The good interrater reliability of the GAF when used by research assistants has been demonstrated in previous studies [29]. In this study, interrater reliability between psychiatrists and the research assistants after training was satisfactory (Intraclass Correlation Coefficient between 60 and 83).

### Clinical Global Impression (CGI) scale

Clinical Global Impression is a three-item scale used to assess treatment response in psychiatric patients. They are: 1) Severity of Illness; 2) Global Improvement; 3) Efficacy Index. Item 1 was used in the study and was rated on a seven-point Likert scale (1 = normal, 2 = borderline mentally ill; 3 = mildly ill; 4 = moderately ill;5 = markedly ill; 6 = severely ill and to 7 = extremely ill). In this paper, we used the severity of illness item. This was rated at the time of assessment, at three months and at six months follow up [32].

Follow up interviews were scheduled by the research assistants at three months and six months from the time of recruitment. This was carried out from the patients’ homes. If a patient was not available at the time of the visit, an appointment would be made and further visits made, up to maximum of three visits after which the patient was declared ‘lost to follow up’.

### Data analysis

The relationship of the symptom severity scales at recruitment and follow up with CGI ratings were calculated to examine the consistency of the scales. Remission of symptoms was determined on the basis of cut-off point of caseness by the specific symptom rating scales.

Outcome was determined by the mean scores of the scales at the three levels of follow up (i.e. at point of first contact, at three and six months). The mean scores were calculated using one way ANOVA for independent samples. The Tukey HSD (Honestly Significant Difference) test was used for Post-ANOVA comparisons. The Tukey HSD test is a post test for pair wise comparisons. A significant F ratio tells us that there are significant differences in the means of the scales. It does not tell whether any particular sample mean is different from any particular other. The Tukey HSD test is recommended for Post-ANOVA comparisons because it is more robust then the t test [33].

To assess factors associated with caseness, analysis was done at bivariate level for three and six months follow up and all variables that were significant qualified for the multivariable logistic regression model for predictors of a case at both three and six months. The level of statistical significance was set at 5% (i.e. P ≤ 0.05).

### Ethical considerations

Ethical clearances were obtained from the following sources: the Human Research and Ethics Committee of Karolinska Institutet in Sweden (Dnr 05/07); the Research and Ethics Committees of Makerere University Medical School (Uganda); the Uganda National Council for Science and Technology Committee on study of Human Subjects (HS 323); the District Health Officers in the districts concerned, the patients and/or their relatives consented to the follow up procedure. Conduct during the study adhered to the Helsinki Declaration [34].

## Results

The sample of 115 participants consisting of 51 Females (44.35%) and 64 males (55.65%), were drawn from 387 attendees at the traditional healers’ shrines or clinics over a six months period. Their specific diagnoses were: Schizophrenia (28, 24.30%); Mania (41, 35.70%); Psychotic depression (46, 40%). The majority were peasants and not in formal employment. The mean age at recruitment was 34.47 (SD 8.19).

**Figure 1:**
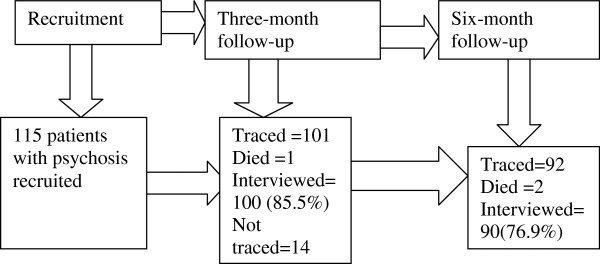
Follow up rates at recruitment and after three and six months.

Figure 1 below shows the follow up rates at recruitment, three and six months.

### Combined use of traditional healing and health centres and general outcome as measured by symptom scales

Table 1 below summarises the general symptom outcome as measured by the PANSS, YMRS and MADRS.

**Table 1:** General outcome of treatment of psychosis by traditional healers at three and six months follow up

**Follow up period**	**Number of non caseness**^**a**^	**Percentage**	**95% CI**
**Baseline**			
Schizophrenia (N=28)	-	24.35	17.43-32.96
Mania (N=41)	-	35.65	27.49-44.76
Depression (N=46)	-	40.00	31.50-49.16
Total			
**3 months:**			
Schizophrenia(N=27)	4	14.81	06.06-32.67
Mania(N=37)	20	54.05	38.30-69.02
Psychotic Depression(N=37)	16	42.24	28.62-59.18
Total	40	39.60	30.61-49.38
**6 months:**			
Schizophrenia(N=26)	8	30.77	16.52-50.18
Mania(N=36)	21	58.33	39.49-70.51
Psychotic depression (N=28)	12	46.43	26.45-61.06
Total	41	45.56	35.64-55.84

^a^ Number of those who scored below the cut off point caseness in the respective scales indicating improvement.

During the three months, most patients with mania (35/37, 94.59%, 95% CI 82.25-98.34) visited both the traditional healers and biomedical services for their symptoms, followed by psychotic depression (30/37, 81.08%, 95%CI 65.67-90.45). Slightly more than half of the patients with schizophrenia (16/27, 59.25%, 95%CI 40.58-75.54) visited both the healer and the hospital. In the six months follow up, the figures dropped by about half for mania and psychotic depression (18/36, 50.00%, 95%CI 34.40-65.60) and (13/28, 46.43%, 95%CI 29.45-64.31), respectively. While for schizophrenia, it more or less remained the same (12/26, 46.13%, 95% CI 28.67-64.67).

### Comparisons of the mean scores of the different rating scales

Table 2 shows a one way Analysis of Variance of independent samples at initial contact, at 3 months and 6 months follow up.

**Table 2:** Results of One way ANOVA showing mean scores of respective rating scales

**Measure**	**Baseline**		**Follow up**					
			**3 months**		**6 months**			
	**Mean score**	**SD**	**Mean score**	**SD**	**Mean score**	**SD**	**Mean Square**	**F P value**
**Positive And Negative Syndrome Scale**								
Total score	123.60	20.90	86.12	21.89	75.23	13.50	375.98	38.59 <.0001
Positive symptoms	28.00	1.20	25.32	1.62	24.01	1.22	1.85	926.42 <0.0001
Negative symptoms	33.10	1.20	22.43	1.30	18.12	1.22	2.73	−308.42 <0.0001
General symptoms	62.50	1.15	39.12	2.80	33.10	1.22	4.42	−615 <0.0001
**Montgomery-Asberg Depression Rating Scale**								
Total score	48.76	4.47	34.62	2.70	27.89	2.28	11.74	276.21 <0.0001
**Young Mania Rating Scale**								
Total score	52.66	5.49	30.83	3.52	24.50	3.46	18.64	278.40 <0.0001
**Clinical Global Impression Severity of Illness**								
score	5.70	0.95	4.26	0.86	3.34	0.81	0.78	188.17 <0/0001
**Global Assessment of functioning**								
Current score	34.08	5.27	50.82	5.94	56.10	8.62	42.92	317.27 <0.0001
**Compass mental health index current well being subscale**								
Current score	6.50	1.42	8.62	1.52	13.40	1.55	2.23	551.71 <0.0001

Tukey HSD test: The absolute difference between any two sample means at HSD [0.05]; HSD [0.01] were all significant, P < 0.01 apart from that of Montgomery-Asberg Depression rating scale for psychotic depression where there was no significant difference between three months and six months.

In terms of percentage reduction in symptoms from baseline, symptom severity improved at 3 and 6 months follow up with PANSS showing a 30% reduction at 3 months and nearly 40% at 6 months overall, 9.57%, 32.23% and 37.41% reduction at 3 months; 14.25%, 45.24% and 47.04% at 6 months on the subscales of positive symptoms, negative symptoms and general psychopathology respectively.

For YMRS symptom reduction was 21% at 3 months and 28% at 6 months. There was a decline for the MADRS scale from 29% at 3 months to 20% at 6 months.

**Figure 2:**
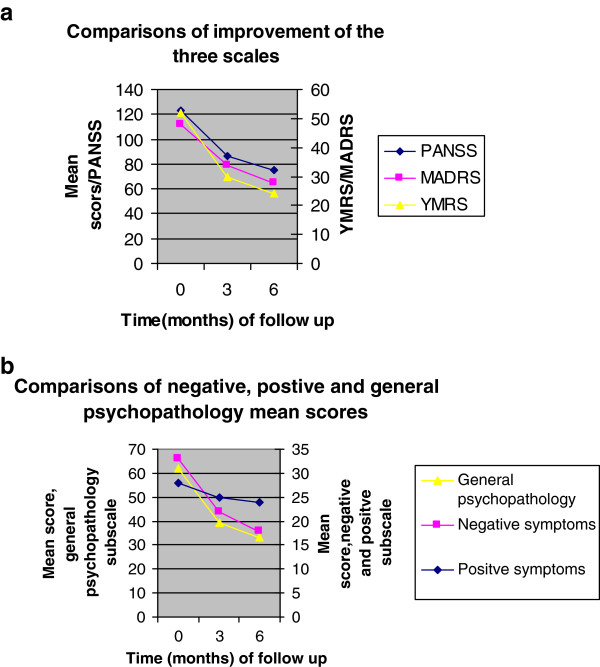
a and b showing comparisons of improvement of PANSS, YMRS and MADRS and positive, negative and general psychopathology scales of PANSS respectively.

Figure 2a and b below shows comparisons of improvement of PANSS, YMRS and MADRS and positive, negative and general psychopathology scales of PANSS respectively.

Table 3 Below shows results of bivariate analysis of factors associated with caseness [35] .

**Table 3:** Shows results of bivariate analysis of factors associated with caseness

	**Three months follow up**					**Six months follow up**				
**Variable**	**Cases N = 61%**	**Non cases N = 40%**	**X**^**2**^	**P value**	**Crude OR (95%CI)**	**Cases N = 49%**	**Non cases N = 41%**	**X**^**2**^	**P value**	**Crude OR (95%CI)**
**Sex**										
Male	50.81	65.00			0.44	55.10	36.59	2.38	0.120	2.13
Female	49.18	35	3.32	0.070	(0.18-1.06)	44.90	63.41			(0.84-5.45)
**Marital status**										
Married	45.90	67.50			0.40	53.06	41.46			1.60
Not Married	54.10	32.50	3.71	0.050	(0.16-1.01)	46.94	58.54	0.78	0.380	(0.64-4.02)
**Employment**										
Employed	47.54	62.50	1.61	0.200	0.54	51.02	43.90			1.33
Not Employed	52.46	37.50			(0.12-1.32)	49.98	56.10	0.21	0.640	(0.53-3.34)
**Economic status**										
Earning < $1	73.77	30.00			6.56	57.14	29.27			3.22
Earning > $1	26.23	70.00	17.09	<0.001	(2.49-17.66)	42.86	70.73	5.94	0.010	(1.23-8.58)
Being in debt	63.93	37.50			2.95	58.18	31.71			3.12
Not being in Debt	36.34	62.50	05.76	0.020	(1.20-7.37)	41.82	68.29	5.71	0.020	(1.20-8.22)
**Age of onset**										
Below 18	67.21	25.00			6.15	61.22	24.39	10.82	0.001	4.89
Above 18	32.79	75.00	15.57	<0.001	(2.32-16.70)	38.78	75.61			(1.79-13.65)
**Duration of symptoms**										
> 3 months	36.07	70.00			0.24	57.14	31.71	5.25	0.020	2.97
< 3 months	63.93	30.00	9.81	0.002	(0.09-0.06)	42.86	70.73			(1.15-7.78)
**Number of episodes**										
2 or more	60.66	37.50			2.57	63.27	39.02	4.33	0.030	2.69
First episode	39.34	62.50	4.30	0.030	(1.05-6.36)	36.73	60.98			(1.05-6.95)
**Family history**										
Positive	68.85	22.50	18.95	<0.001	7.61	59.18	29.27			3.50
Negative	31.15	77.50			(2.79-21.36)	40.82	70.73	6.89	0.008	(1.33-9.36)
**co morbidity**										
2 or more	62.30)	25.00			4.96	59.18	29.27			3.50
Only disorder	37.70	75.00	12.08	0.001	(1.89-13.28)	40.82	70.73	6.89	0.008	(1.33-9.36)
**Severity(GAF)**										
Mod-severe	65.57	32.50	9.31	0.002	(1.57-10.13)	51.02	29.27	3.51	0.060	(0.96-6.66)
Mild	34.43	67.50			3.96	48.98	70.73			2.52
**Treatment:**										
Both	68.85	97.50	10.75	0.001	0.06	77.55	12.20	35.64	<0.001	24.87
Only T/H or WM	31.15	2.50			( 0.00-0.43)	22.45	87.80			(7.03-94.84)

In multivariate analysis, only two variables, namely being in a worrisome debt and combined use of biomedical services and traditional healing remained significant at both 3 and 6 months follow up. At 3 months follow up, those who combined treatment were less likely to be cases (P = 0.002; OR 0.26 [0.15-0.58]), while at 6 months follow up, they were more likely to be cases (P = 0.020; OR 2.05 [1.10-3.19]). Being in a worrisome debt was associated with cases at both 3 and 6 months. (P <0.001; OR 11.21 [6.58-17.26]) and P =0.01; OR = 2.23[1.41-3.53] respectively.

## Discussion

This study is a follow up of a cohort of patients with severe mental illness attending traditional healers. Follow up was conducted at both three and six months for several reasons. First, the three months follow up was important to reduce the risk of missing out participants whose symptoms could have remitted within this three months [11,36]. Secondly to avoid high attrition rate and finally, a six months of a follow up period was considered valuable for continued assessment of patients whose symptoms had potential for remitting given a longer treatment period.

In light of the local context were patients ability to return for review are a function of various factors such as lack of transport, the follow up rate of 85.5% at three months and 76.6% at six months was satisfactory. There was no significant difference in key baseline socio-demographic or clinical variables between those who were successfully traced and those lost to follow up.

### Key findings

There was a general trend of reduction in symptom scales at 3 and 6 months follow up. The percentage reduction was greatest in the PANSS scale followed by YMRS, and MADRS scale. However, in terms of dichotomous caseness, patients with mania had most recovery followed by patients with psychotic depression and lastly patients with schizophrenia.

Over 80% of the subjects used traditional healing and biomedical services concurrently with the majority being patients with mania. The greatest concurrent use occurred in the first three months of follow up. At bivariate level of analysis, poor economic status, early age of onset, longer duration of symptoms, previous episodes, positive family history, current illness severity, co morbidity and combined use of biomedical services and traditional healing were significantly associated with outcome of psychosis. At multivariate level, only combined use of biomedical services and traditional healing and being in debt remained significant.

To our knowledge, there are hardly any controlled studies of outcome of treatment of patients with severe mental illness who use traditional healing practices. Contemporary biomedical models which most health workers subscribe to, equate health and sickness with normal and abnormal biological functioning respectively. This approach assumes that symptoms can be adequately characterized in terms of measurable changes in the basic biological processes in the human body and reduces all health or illnesses phenomena to these processes [37]. By extension, conventional western medicine argues that human mind can be described in terms of neurobiology. Biomedical psychiatry has endorsed this model and the corollary that normal pathology states of the mind are reduced to basic neuropsychological or neuroanatomical processes. In this broad context, it has been argued that the claims of traditional healing practices are of capricious and lacks scientific rigor, diagnostic accuracy and that outcome measures are not clear or objective. Lack of control measures in those studies is stated as another drawback [37].

In this study, we attempted to overcome these problems. The patients were carefully diagnosed according to DSM IV diagnostic criteria by using the MINI plus. In addition to use of symptom outcome measures, we used CGI and GAF as objective measures of impairment and the study was prospective. The principal difficulty and limitation in this study, however, was that we could not control the subjects. At the time of the study, no sensible or ethical solution to the problem of control of the subjects was evident to us. Traditional healing practices are not officially accepted as a form of treatment for severe mental illnesses although 4 in 5 Ugandans visit traditional healers [38]. On the other hand, there is concern that controlled trials may not capture the full richness and diversity of traditional healing practices and that randomisation may undermine the representation of the therapeutic encounter [39]. Attempts to compare those patients who go to traditional healing practices with those who go for biomedical services were limited by the design of the study. On the other hand, since severe mental illnesses are relatively enduring, comparisons between different periods of the patients’ lives offer a suitable means of obtaining a control. Thus, comparisons were made at three and six months with first contact with the patient.

Of particular interest in the present study is the finding that patients who combined biomedical services and traditional healing were less likely to be cases at three months follow up but more likely to be cases at six months follow up. Alternative explanations for the improvement at three months did not seem plausible. Those that were considered included spontaneous remission, selective attrition and a combination of use of western health facility and traditional healing.

Spontaneous remission seemed an unlikely explanation for the results. All patients were diagnosed with MINI plus for DSM-IV diagnosis thus eliminating acute psychoses. For affective psychosis, spontaneous remission may occur between 3 to 6 months. This study registered a positive change at 3 months, but not at 6 months.

A possible explanation for improvement of our patients might be selective patient attrition. There was no evidence that the more disturbed patients had dropped out of the study. Comparisons were made between the patients who completed the study and those who dropped out. No significant differences were found on any measure. There was a tendency for the drop outs to be females, born in Busoga but married in another district with a diagnosis of mania. However, this did not reach statistical significance. Furthermore; the drop out rate was low at 14.50% at three and 23.1% at six months.

Combination treatment seems to be the most plausible explanation for the improvement at 3 months.

An increasing number of studies have shown that psychological treatment combined with medication work additively on different complementary aspects of illness resulting in clinical benefits over medication alone [40-42]. Psychological treatment helps social functioning whereas medication controls abnormalities of mood and thought [43]. Although in our study we did not include the kind of treatment these patients got from the traditional healers, previous research reports have indicated that traditional healers are good at dealing with psychosocial issues, thus offering psychological aspects [44,45].

At six months follow up, combined use of both biomedical services and traditional healing was more likely in the cases. This may be explained by the need to achieve improvement by those who had not yet registered it. Hence the continued use of both biomedical services and traditional healing.

Our finding of over 20% to nearly 40% reduction in symptom scales scores is higher than a similar study carried out in India that reported 18.90% reduction in symptoms. The authors attributed their observed clinical improvement to the cultural power of residency in the healing temple and a supportive, non threatening and a reassuring setting since their patients had not had any western treatment [46]. It could be that our patients had both medications from the western health facilities and the psychosocial input from the traditional healers thus offering better outcome.

Although ours is the first study to use standard clinical assessments to try and evaluate the outcome of traditional healing practices, our findings are only suggestive and not conclusive owing to the limitations of our methods and therefore the results should be interpreted with caution. First the numbers of individual psychotic illnesses were few. This could have exaggerated the percentage reduction. However, the number of 20 people is adequate for a statistically significant measuring of the difference before compared to after the intervention [47].

Secondly, it was not possible for us to have patients go for only traditional healing or western facility since in reality both are used concurrently [48,49] .This makes it hard to maximise methodological rigour and minimise the intrusiveness of the research [46] .

Despite efforts to explain the improvement in our patients, the other possible explanations like natural course of schizophrenia and mania may still be the reason for the improvements that was seen. A longer-term follow-up, at least for 12 months with a larger sample size would help to address these possible explanations in order to have a more conclusive report about the effects of traditional healing in psychosis.

Nevertheless, such a research has a useful role in helping to assess needs and resources for developing locally relevant mental health programmes [46]. In terms of research, a lot remains to be done in this area of traditional healing in relation to psychosis.

## Abbreviations

WHO: World Health Organisation; DSM-IV: Diagnostic and statistical manual, fourth edition; CWM: Conventional Western medicine; TH: Traditional healers.

## Competing interest

The Authors declare that they have no competing interests.

## Authors’ contribution

At the time of the study, the first author, CA was a PhD student being supervised by SE, ESO, PW and SM. CA supervised data collection, participated in data analysis and drafted the manuscript. All authors participated in the study from its inception, read through and approved manuscript.

## Authors’ information

Catherine Abbo, MD, PhD is lecturer and psychiatrist in the Department of Psychiatry. She completed her PhD study from Karolinska Institutet, Department of Clinical Neuroscience, Section of Psychiatry, Stockholm, Sweden and Makerere University, Department of Psychiatry.
